# Ca Distribution Pattern in Litchi Fruit and Pedicel and Impact of Ca Channel Inhibitor, La^3+^

**DOI:** 10.3389/fpls.2017.02228

**Published:** 2018-01-09

**Authors:** Wen-Pei Song, Wei Chen, Jun-Wen Yi, Hui-Cong Wang, Xu-Ming Huang

**Affiliations:** Physiological Laboratory for South China Fruits, College of Horticulture, South China Agricultural University, Guangzhou, China

**Keywords:** calcium transport, pedicel, pericarp, Ca oxalate, phloem pathway

## Abstract

Calcium (Ca) deficiency in fruit causes various physiological disorders leading to quality loss. However, disorders related to Ca deficiency are not simply caused by a shortage of calcium supply. Ca distribution is also an important relation. This study examined Ca distribution pattern in fruit and pedicel in litchi (*Litchi chinensis* Sonn.) and the influence of Ca channel inhibitor La^3+^ on fruit Ca uptake and distribution. *In situ* distribution of Ca in the phloem and xylem tissues of the pedicel was visualized by Ca mapping with X-ray microanalyzer. Ca^2+^ analogy Sr^2+^ was used to trace Ca^2+^ transport pathway to fruit as well as distribution pattern. The results showed Ca was more distributed in the pericarp, especially the distal part. Ca level in the bark/phloem was always significantly higher than in the xylem and increased with stem age, suggesting constant influx of Ca into the phloem from the xylem. La^3+^ increased the ratio of Ca in the xylem to that in the bark in the pedicel and significantly reduced Ca accumulation by 55.6% in fruit, suggesting influx of Ca into the symplast was involved in fruit Ca uptake. Sr^2+^ introduced from fruit stalk was found to be transported to fruit through the phloem as Sr was largely distributed in the phloem, and fruit stalk girdling significantly reduced Sr accumulation in the pericarp. Ca mapping across the pedicel revealed Ca-rich sites in the parenchyma cells in the phloem and along the cambium, where abundant Ca oxalate crystals were found. The results suggested extensive influx of Ca from xylem/apoplast pathway into the phloem/symplast pathway in the pedicel, which enables phloem/symplast pathway to contribute a considerable part to Ca uptake in litchi fruit.

## Introduction

Calcium (Ca) is one of the essential nutrients in plants, serving as an important element for cell wall construction and membrane stabilization, as an intracellular messenger for signaling and regulations, and as a counter ion for organic acids (White and Broadley, 2003; Hepler, 2005). Deficiency of this element reduces storage quality and causes a number of fruit disorders including bitter pit, blossom-end rot, water soaking, and fruit cracking (Shear, 1975; White and Broadley, 2003; Huang et al., 2005). Very limited amount of Ca can be supplied to fruit by spraying, which more often than not fails to eradicate the problems of Ca deficiency (Himelrick and Ingle, 1981; Ferguson and Watkin, 1989; Saure, 2005; Zhong et al., 2006; Huang et al., 2008). Therefore, the continuous uptake of Ca by fruit depends on the tree supply, i.e., root absorbed Ca delivered to up-ground parts via the long-distance vessel transport system. However, Ca allocation among tissues, between symplastic and apoplastic spaces, or among various cell compartments also determines the availability Ca for its structural function (Tonetto de Freitas and Mitcham, 2012), failure of which induced various Ca deficiency symptoms, such as blossom end rot in tomato (Tonetto de Freitas et al., 2014).

Calcium transport is generally believed to be exclusively through the xylem/apoplast pathway because Ca is considered relatively immobile in the phloem/symplast system (Ferguson, 1980; Hanson, 1982; Zocchi and Mignani, 1995; Saure, 2005; Hocking et al., 2016). Therefore, it is well accepted that xylem flow is the exclusive contributor of Ca to fruit. In some fruits, such as apple (Tagliavini et al., 2000; Miqueloto et al., 2014) and kiwifruit (Dichio et al., 2003; Morandi et al., 2010), xylem-contributed sap influx to fruit is lost due to xylem functionality loss during the late fruit development, which causes reduction of Ca uptake (Dichio et al., 2003; Miqueloto et al., 2014). However, Jones et al. (1983) found great difference between estimated calcium accumulation based on vessel influx volume and calcium concentration and measured calcium accumulation in apple fruit, indicating phloem might also be a major pathway for calcium transport. There are also evidences supporting the involvement of phloem in Ca transport in apple (Stebbins and Dewey, 1972; Himelrick and McDuffie, 1983) and pistachio (Vemmos, 2005). Yang and Jie (2005) suggested that both symplast and apoplast pathways participated in Ca movement into fruit. Hence, the pathway(s) of Ca transport to fruit is still a matter of dispute and might be different among plant species.

Litchi (*Litchi chinensis* Sonn.) is a commercially important tropical crop that bears arillate fruit in panicles produced from the terminal shoots. In our previous study with litchi (Huang et al., 2006), X-ray microanalysis displayed a far greater abundance of Ca in the pedicel than in the pericarp, and we thus hypothesized that there was a “bottleneck” in Ca transport to fruit in the pedicel. In this study, we further observed calcium distribution in pedicel tissues and tried to understand relation of pedicel calcium accumulation to fruit calcium accumulation, results from which strongly suggested the involvement of the phloem/symplast pathway in fruit calcium uptake.

## Materials and Methods

### Materials

Unless otherwise specified, the experiments were carried out with air-layered ‘Huaizhi’ litchi (*L. chinensis* Sonn.) trees at ages of 12–14 years cultivated in the demonstration orchard at Dongguan Agricultural Research Center, Dongguan, China, or in the experimental orchard at South China Agricultural University, Guangzhou, China.

### Ca in Different Parts of the Fruit and Stalks

**FIGURE 1:**
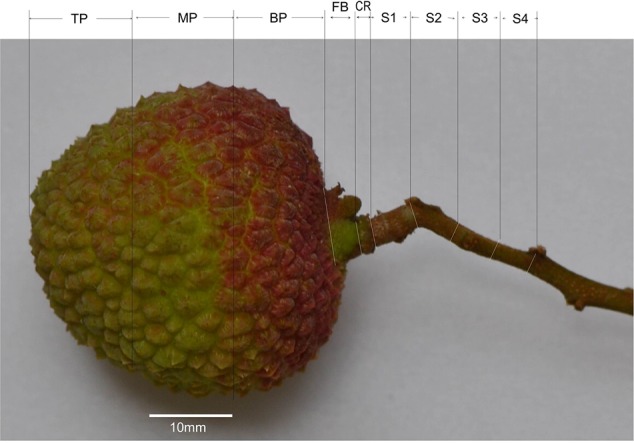
Sections of a mature litchi fruit with its stalk for measurements of calcium (Ca) concentrations. TP, the top 1/3 fraction of the pericarp; MP, the middle 1/3 fraction of the pericarp; BP, the basal 1/3 fraction of the pericarp; FB, fruit base; CR, calyx ring (about 1 mm in length); S1, 0–5 mm segment of the fruit stalk (basically the pedicel); S2, 5–10 mm segment of the fruit stalk; S3, 10–15 mm segment of the fruit stalk; S4, 15–20 mm segment of the fruit stalk.

Red mature fruit (76 days after anthesis, DAA) along with their stalks (including the pedicle) were sampled randomly from the same bearing shoots in the same tree. The fruit samples were sectioned into the top pericarp (top 1/3 of fruit length, TP), the middle pericarp (central 1/3 of fruit length, MP), the basal pericarp (basal 1/3 of fruit length, BP), fruit base (the knob-like end of the fruit adjoining the calyx and the pericarp base, FB), calyx ring (C), and 5-mm segments of fruit stalk (including the pedicel), which were indicated by S1, S2, S3, and S4 according to their distance from the calyx ring (**Figure 1**). The corresponding sections from 10 randomly selected fruit were pooled as one replicate and oven dried at 65°C for 48 h, and their dry weights were collected before Ca analysis. The dried tissues were ground into powder. The powder was transferred to a 10 ml melting pot, burnt on an electric stove until smoking stopped, and ashed in an ashing furnace at 550°C for 5 h. The ash was dissolved with 0.1 N HCl solution and set to 25 ml for measuring Ca concentration using a Hitachi Z-5000 atomic absorption spectrometer. The analysis was conducted with five replicates (*n* = 5).

### Ca in the Pericarp, and in the Bark and Wood (Xylem) in Stems of Different Ages

Bark (chiefly phloem and cortex) and wood (xylem tissue) from the limbs (>6 cm diameter), large branches (4 cm diameter), small branches (2 cm diameter), shoots (0.8 cm diameter), and fruit stalks, as well as the entire pericarp, were separately collected at 76 DAA, when fruit were fully matured. Bark pieces of 1 cm × 1 cm were directly cut out from the branches, and wood tissues from them were drilled out from a depth of 1 cm using a hand-carried driller equipped with a 0.6 cm drilling head. Half-circle bark from shoots was peeled off at a width of 2 cm, and the exposed wood tissue was cut off. Fruit stalks were separated into bark and wood. The tissues, except for those from the fruit stalk, were oven dried at 65°C for 48 h and ground into powder before Ca analysis as above mentioned. Fresh bark and wood tissues of fruit stalks were prepared according to the procedures of Huang et al. (2006) modified from Chen and Uemoto (1976) for measurements of water-soluble Ca, wall-bound structural Ca and Ca in oxalate. A 0.5 g tissue sample was ground in 10 ml distilled water and the homogenate was centrifuged at 5000 g for 10 min at room temperatures (27°C). The sediment was suspended in 10 ml distilled water and centrifuged again at 5000 *g* for 10 min. The supernatant with soluble Ca from the two centrifuges were pooled and set to 25 ml. The sediment was resuspended in 10 ml 2% (v/v) solution of acetic acid to release water insoluble structural calcium, and centrifuged at 5000 *g* for 10 min. The sediment was re-suspended in 10 ml of the acetic acid solution and centrifuged again. The pooled supernatant from the two centrifuges was set to 25 ml and used as structural Ca extract. The sediment was then suspended in 25 ml of 1% hydrogen chloride solution that dissolves calcium oxalate. After centrifuge at 5000 *g* for 10 min, the supernatant was collected. The calcium content in different fractions was measured with an atomic absorption spectrometer (Hitachi Z-5000) using 1, 2, 5, and 10 mg l^-1^ CaCl_2_ in distilled water, 2% (v/v) glacial acetic acid solution and 1% hydrogen chloride solution as standards for water soluble Ca, structural Ca and Ca oxalate, respectively. Samples from three trees (*n* = 3) were analyzed.

### Effect of Girdling Made on Bearing Shoot on Ca Uptake in Fruit

The experiment was carried out with 12 year old trees of ‘Nuomici’ litchi. Five pairs of bearing shoots around 1 cm in thickness with similar foliage and fruit set were chosen from “Y” branches at different canopy positions of one selected tree. One of the bearing shoots in each pair received no treatment, while the other was given girdling treatment, which was carried out 14 DAA at 80 cm from the panicle by circled bark stripping at a width of 3 mm. Eight weeks later, final fruit set was recorded for each bearing shoot and the mature fruit samples were collected, individually weighed, and dissected into pericarp, seed and aril. After recording the pericarp weight, the pericarp from all the fruit in the sample shoot were pooled as one replicate and used for Ca analysis, which was carried out with five replicates from five bearing shoots.

### Ca Mapping of Litchi Tissues Using Electron Probe Microanalyzer

Sample preparation and electron probe analysis were performed based on the method of Song et al. (2014). Briefly, fruit stalk, pedicel or fruit base were cut into 0.5-mm thick sections, and stuck onto a copper stand using electrically conductive glue and coated with platinum for 90 s in a JFC-1600 auto fine coater. The prepared samples were observed and analyzed using a JXA-8100 electron probe microanalyzer. All samples were kept at a fixed working distance of 11 mm from the probe sensor, and the electron probe used an accelerating voltage of 20 kv and an emission current of 2 × 10^-8^ A. Distribution of Ca in tissues was mapped and visualized by computer with white spots on a black background, where a higher Ca level was indicated by denser spots. After the Ca mapping image was collected, the secondary electron image of the tissue was captured. Ca mapping photos were processed with Photoshop to remove the black background, and the white “Ca spots” were converted to red spots and then combined with the corresponding secondary electron images of the tissues. These combined images gave direct views of Ca distribution in the tissues.

### Microscopic Observation of Calcium Oxalate Crystals in the Phloem Tissue of the Pedicel

The bark tissues were dissected from the pedicel and cut into pieces of 1.0 mm^3^ and fixed in formalin-acetic acid-alcohol fixative (70% alcohol: acetic acid: formalin = 18:1:1, v/v/v) at room temperature for 24 h. The fixed samples were dehydrated in a series of ethanol solutions (70, 85, 95, 100, 100, and 100% [v/v]) for 15–20 min at each concentration, then in 50% (v/v) ethanol: 50% (v/v) propylene oxide for 15 min, and finally in 100% propylene oxide for 15 min. The samples were then transferred subsequently into 1:1 (v/v) and 1:3 (v/v) propylene oxide: Epon 812 epoxy resin mixtures each for 12 h, and then submerged in pure Epon 812 epoxy resin for 12 h. Hereafter, the samples were embedded in pure Epon 812 epoxy resin, which were allowed to be polymerized at 40° and then 60°C each for 24 h. Sample blocks were sectioned on an ultramicrotome (Reichert-Jung Co., Veina, Austria) with glass blades. Semi-thin sections of 2 μm in thickness were stained for 1 min with 0.05% toluidine blue O at 60°C and observed under a Leica DMLB light microscope.

### Introducing Calcium Channel Blocker La^3+^ to Fruit

La^3+^ is a strong non-selective Ca channel blocker and has been used to examine the involvement of Ca channels in Ca uptake and cell functions (Lewis and Spalding, 1998; Demidchik et al., 2002). To test the effect of La^3+^ on Ca uptake by fruit, we introduced a 0.1% (w/v) LaCl_3_ solution through the bearing shoots. The treatment was made at 30 DAA. Two 0.7 to 0.8 cm thick bearing shoots growing toward the same direction but separated from each other by at least two orders of branching were selected from each of five different directions of the canopy of a selected ‘Huaizhi’ tree. A hole of 2 mm in diameter was drilled across the shoot stem about 60 cm from the terminal fruit cluster with a battery-powered drill. One end of a piece of cotton string wetted with a 0.1% (w/v) LaCl_3_ solution was inserted into the hole, while the other end of the string was placed into a 10 ml tube filled with the solution. The tube was refilled every day to replace the volume drawn away by the transpiring shoot to keep the string constantly wet. For control, the bearing shoot was processed in exactly the same way but the 0.1% (w/v) LaCl_3_ solution was replaced by distilled water. Fruit were sampled on the day of treatment and at full maturity (76 DAA) for dry mass and calcium analysis. In the second sampling, bark and wood tissues from the fruit stalk, pericarp, flesh, and seed were separated and oven dried at 65°C for 48 h. The dried tissues were used for Ca analysis. The experiment was conducted with five replicates using the bearing shoot as the experimental unit. Meanwhile, five fruit with a pedicel each from La^3+^ treatment and control were used for Ca mapping analyses.

### Tracing Sr^2+^ Fed to Fruit through Stalks

The strontium ion (Sr^2+^) is an analog of Ca^2+^ and has been found to be transported and distributed in the same pattern as Ca^2+^, and therefore strontium has been widely used as a calcium transport pathway tracer in plants (Laszlo, 1994; Storey and Leigh, 2004; Rosen et al., 2006). In this study, we fed Sr^2+^ through fruit stalks, intact or girdled, and observed Sr distribution in the pedicel and the pericarp using the above mentioned electron probe microanalyzer. Twelve fruit with stalks were harvested during ripening at 70 DAA. Stalks of six fruit were girdled at a length of 4 cm from the basal end, leaving at least 3 cm of ungirdled section beneath the fruit. To prevent air embolism, the girdled stalks were cut in distilled water by about 0.5 cm and immediately inserted into a solution of 5% (w/v) strontium nitrate and the upper end of the girdling was kept about 0.5 cm above the solution surface. The remaining six fruit were without girdling. Their intact stalks were inserted into 5% (w/v) strontium nitrate solution after an end-cut in distilled water leaving a similar length to the girdled ones. Fruit were allowed to take in Sr^2+^ through the fruit stalk for 24 h at ambient conditions (temperature around 28°C, RH around 82%). Then the pedicel and the pericarp were sampled for Sr distribution analysis (Sr mapping) using a JXA-8100 electron probe microanalyzer. The sample processing and working conditions of the microanalyzer were the same as for Ca mapping. In addition, energy diffraction spectra of micro-areas of 0.005 mm^2^ in the xylem and phloem of the pedicel and in the pericarp were each collected for 60 s at an accelerating voltage of 20 kv and an emission current of 2 × 10^-9^ A. The mass percentage of Sr was calculated relative to the total mass of C, O, K, Sr, and Pt. The experiment was with six replications using tissue samples from six fruit.

### Statistics

The experiments carried out with a randomized design with number of replicates indicated in each analysis mentioned above. SPSS 10.0 (SPSS Inc., Chicago, IL, United States) was used to conduct LSD multiple arrange tests and *t*-tests, and graphs were generated with Excel 2003.

## Results

### Ca Distribution Pattern in the Pericarp and Fruit Stalk

**FIGURE 2:**
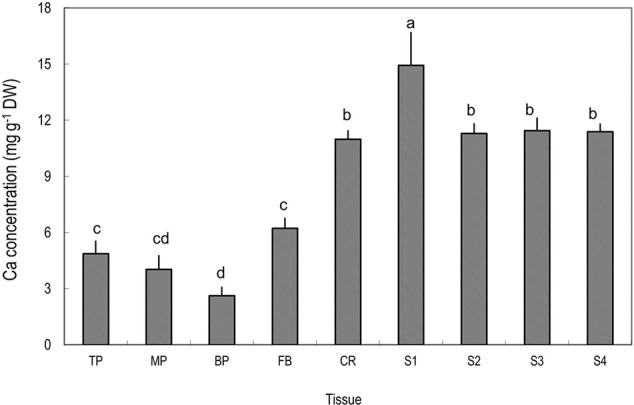
Calcium concentrations in different tissues of mature litchi fruit. Tissues represented by the labels on the X axial refer to **Figure 1**. Different letters above columns indicate significant difference among different tissues (*P* < 0.05, LSD, *n* = 5). Vertical bars represent standard errors of means.

Among the tissues tested, pericarp had the lowest Ca concentrations (**Figure 2**). Within the pericarp, the basal fraction (BP) had the lowest Ca level, while the top/distal fraction (TP) had the highest Ca level. The fruit base (FB) had a Ca concentration significantly higher than the middle and basal pericarp but significantly lower than any part of the fruit stalk. The highest concentration of Ca was found in the segment of the fruit stalk adjoining the fruit base, i.e., S1, consisting chiefly of the pedicel, where Ca concentration was three to five folds higher than in the pericarp sections (**Figure 2**). Sections of fruit stalk further from the fruit had lower Ca concentrations than S1.

### Ca in the Bark and Wood (Xylem) Tissues in Stems of Different Ages

**FIGURE 3:**
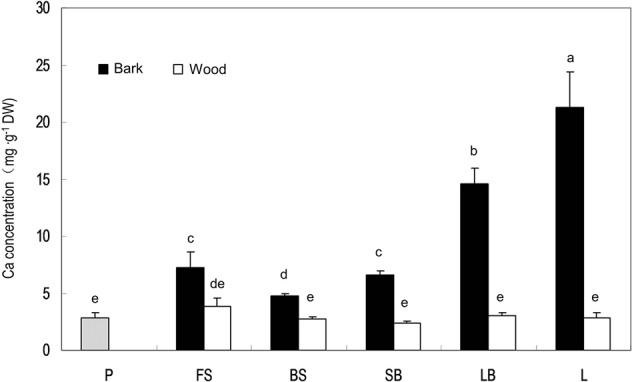
Calcium concentrations in the pericarp of a mature fruit and in the bark and wood tissues of stems of different ages. P, pericarp; FS, fruit stalk; BS, bearing shoot; SB, small branch (2 cm diameter); LB, large branch (4 cm diameter); L, limb (>6 cm). Different letters indicate significant differences among tissues (*P* < 0.05, LSD, *n* = 3).

Stem age is reflected by distance from the terminal fruits in litchi. Ca concentration in stems was always higher in the bark than in the wood/xylem and the pericarp (**Figure 3**). There was no significant difference in Ca concentration in the xylem among different-aged stems, whereas older stems tended to have higher Ca concentrations in the bark than younger stems, suggesting that phloem and cortex tissues constantly accumulate Ca, while xylem tissues do not. However, the stem age pattern of Ca in the bark was broken in the fruit stalk because this youngest stem closest to the fruit had a higher Ca concentration than the older bearing shoot produced in the previous season (**Figure 3**).

### Ca Mapping in the Fruit Stalks

**FIGURE 4:**
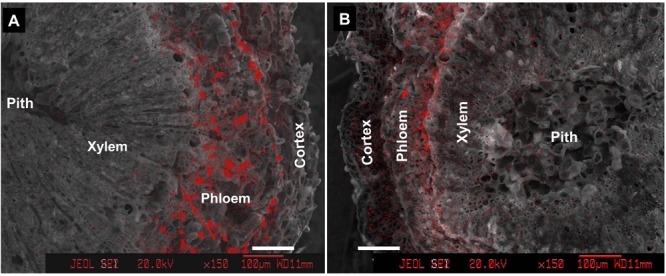
Calcium distribution in cross sections of pedicel (S1) **(A)** and fruit stalk below the pedicel (S2) **(B)**. Bars = 100 μm.

Within the pedicel (S1) and fruit stalk below the pedicel (S2), Ca was much less distributed in the xylem compared with the outer phloem and cortex tissues (**Figure 4**). Phloem had the greatest abundance of calcium among all the tissues within the fruit stalk. In the pedicel (**Figure 4A**), a large number of Ca-rich bodies could be found in the entire phloem tissue, while in the fruit stalk further from the fruit, calcium-rich bodies was smaller and more distributed in the inner phloem adjacent to the xylem (**Figure 4B**). Obviously, Ca was more abundant in the pedicel than in the distant fruit stalk, agreeing with the results shown in **Figure 2**.

### Ca Forms in the Pedicel

**FIGURE 5:**
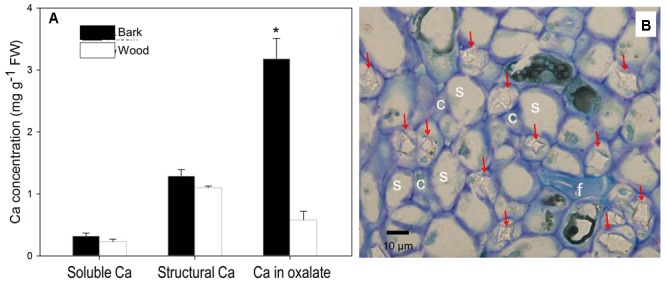
Contents of different forms of Ca in the bark and wood of litchi fruit stalk **(A)** and presence of calcium oxalate crystals in the phloem **(B)**. Arrows in subfigure **(B)** stands for crystals of Ca oxalate; s, c, and f for sieve element, company cell and fiber, respectively. Asterisk indicates significant difference between tissues at *P* < 0.05, *t*-test. Bar = 10 μm.

We further dissected fruit stalk into wood consisting chiefly of xylem and bark including the phloem and the cortex, and measured Ca in different forms in the two parts (**Figure 5A**). Concentrations of soluble Ca and structural Ca were slightly higher in the bark but no significant different between the two part. However, Ca in oxalate in the bark, representing 66% of Ca in the tissue, was significantly higher than in the wood (**Figure 5A**). Ca in oxalate contributed the major difference in Ca concentration difference between the bark and the wood. Indeed, within the phloem, a large number of Ca crystals were observed inside the parenchyma cells surrounding the sieve elements (**Figure 5B**).

### Effect of Bearing Shoot Girdling on Fruit Ca Uptake

Shoot girdling significantly increased fruit retention (7.3 vs. 1.3 fruit per panicle) but significantly reduced fruit size (9.0 g vs. 18.1 g). Compared with the control, the treatment significantly reduced pericarp calcium concentration and pericarp Ca uptake (1.62 mg fruit^-1^ vs. 3.60 mg fruit^-1^, **Table 1**). The results suggest that phloem transport pathway, which is cut off by girdling treatment, might contribute to calcium uptake in litchi fruit.

**Table 1:** Effect of girdling made on bearing shoot on fruit size, number and Ca uptake in ‘Nuomici.’

Treatment	Fruit weight (g)	Pericarp weight (g)	Fruit number per panicle	Pericarp Ca concentration (mg g^-1^ fw)	Total Ca in pericarp (mg fruit^-1^)
Control	18.1	5.0	1.3	0.64	3.60
Girdling	9.0^∗^	3.1^∗^	7.3^∗^	0.36^∗^	1.62^∗^

*Asterisk indicates significant difference at *P* < 0.05, *t*-test.*

### Effect of La^3+^ on Ca Accumulation in Fruit and Stems

**FIGURE 6:**
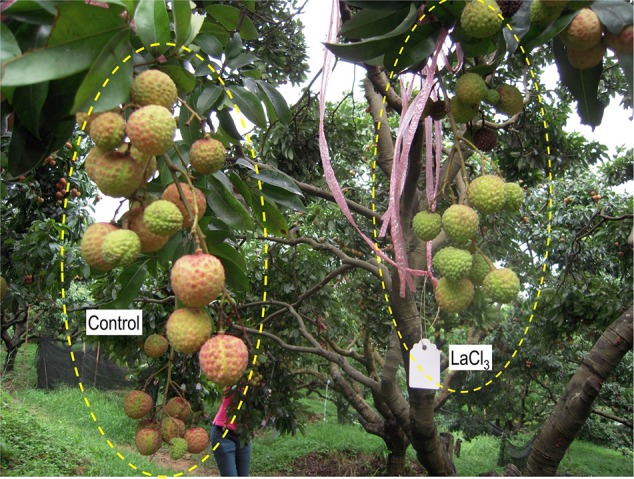
On-tree fruit clusters treated with water (CK) and LaCl_3_ solution. Note that fruit treated with LaCl_3_ were smaller and poorly colored compared with the control fruit. Photo was taken 69 DAA or 39 days of LaCl_3_ treatment.

**FIGURE 7:**
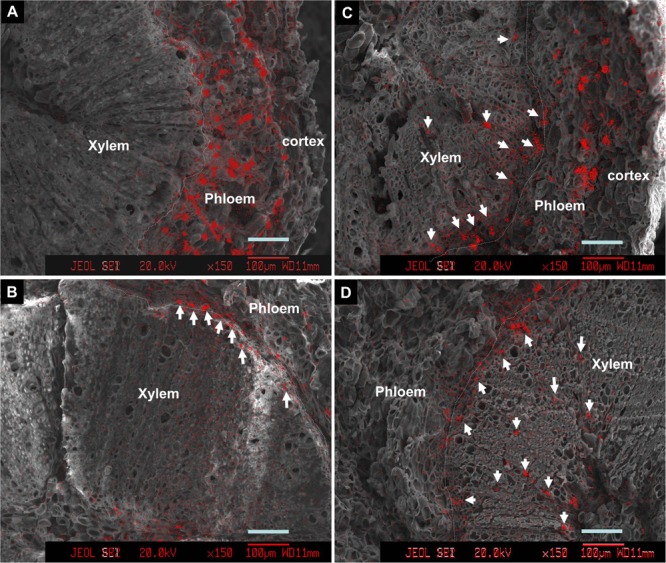
Calcium distribution in cross sections of pedicels **(A,C)** and fruit base **(B,D)** in the control **(A,B)** and the La^3+^ treatment **(C,D)**. Samples were taken 76 DAA or 46 days of treatment. Arrows indicate Ca-rich sites in the xylem tissue. Dashed lines are the borderlines between phloem/cortex and xylem (cambium). Bars = 100 μm.

Introduction of ion channel blocker La^3+^ to fruit through the bearing shoot significantly reduced fruit size (**Table 2**) and inhibited coloration (**Figure 6**). The treatment significantly decreased Ca accumulation by 55.6% in fruit. The decrease was chiefly from a Ca decrease in the pericarp because Ca content in the seed and the flesh were low and similar in the control and treatment (**Table 1**). La^3+^ treatment significantly increased Ca concentrations in the fruit base (**Table 3**). In the fruit stalk, the treatment slightly decreased Ca in the bark but increased it in the wood (**Table 3**). The ratio of Ca concentration in the bark to that in the wood in the fruit stalk decreased from 1.64:1 in the control to 1.15:1 in the treatment. Ca mapping in the pedicel (S1) and fruit base (FB) showed that after La^3+^ treatment the abundance of Ca in the xylem tissue increased, while that in the phloem or cortex was reduced (**Figure 7**). More Ca remained on the xylem side, especially along the xylem/phloem “borderline” (**Figures 7C,D**).

**Table 2:** Effects of La^3+^ on fruit growth and calcium accumulation in litchi.

Days after treatment		Fruit weight (g)	Total Ca (mg fruit^-1^)	Pericarp Ca (mg fruit^-1^)	Flesh Ca (mg fruit^-1^)	Seed Ca (mg fruit^-1^)
0		0.36	0.55	–	–	–
46	CK	11.7^a^	3.32^a^	2.19^a^	0.46^a^	0.67^a^
	La^3+^	8.2^b^	1.78^b^	0.95^b^	0.38^a^	0.45^a^

*Fruit samples of day 0 were too small to be separated into flesh, seed, and pericarp tissue, so the entire fruit was used to determine Ca content. At full maturity (day 46), fruit samples were separated into pericarp, seed, and flesh tissue. Fruit Ca was the sum of the calcium content in the three parts. CK stands for the control. Different letters following means of day 46 indicate significant differences between the control and La^*3*+^ treatment (*P* < 0.05, pairwise *t*-test, *n* = 5).*

**Table 3:** Effects of La^3+^ on calcium concentrations in the pericarp, fruit base, bark, and wood tissue of pedicel.

Treatment	Ca concentration (mg g^-1^ DW)		
	Pericarp	Fruit base	Pedicel		
			Bark	Wood	Bark/wood ratio
CK	3.6^a^	3.7^b^	6.9^a^	4.2^b^	1.64
La^3+^	2.3^b^	4.5^a^	6.1^a^	5.3^a^	1.15

*Different letters following data of day 46 indicate significant differences between the control and La^*3*+^ treatment (*P* < 0.05, pair-wise *t*-test, *n* = 5).*

### Tracing Translocation of Sr Introduced through Fruit Stalks

**FIGURE 8:**
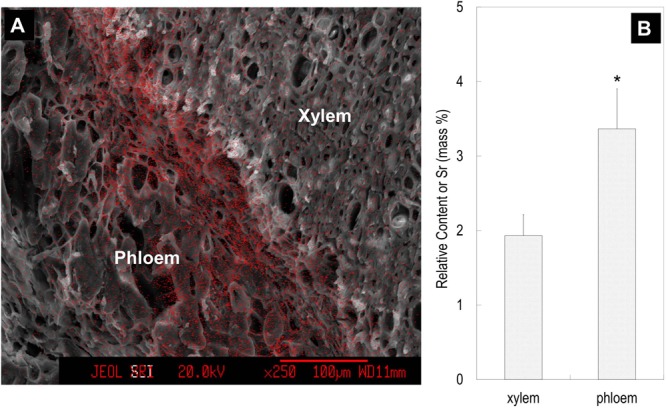
Strontium ion mapping in the pedicel **(A)** and the relative contents of Sr in the phloem and xylem **(B)**. Relative content of Sr was the mass percentage of Sr relative to the total mass of C, O, K, Sr, and Pt. Fruit samples were taken 76 DAA. Asterisk indicates significant difference at *p* < 0.05, *t*-test, *n* = 6. Bars = 100 μm.

**FIGURE 9:**
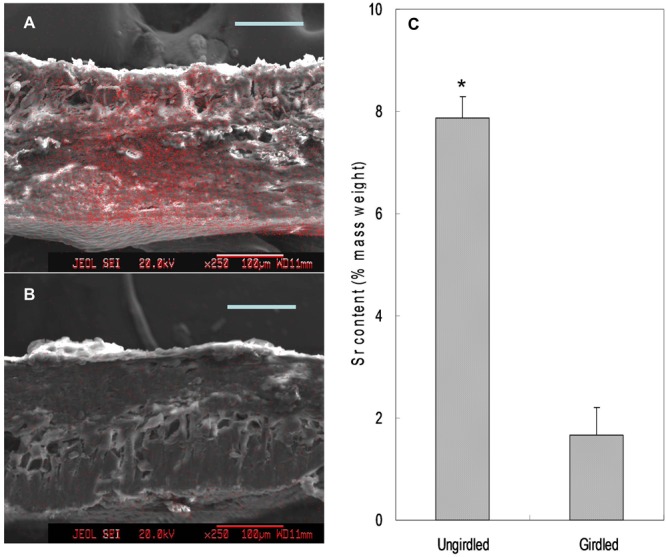
Mapping **(A,B)** and relative contents **(C)** of Sr in the pericarp. Relative Sr content was the mass percentage of Sr relative to the total mass of C, O, K, Sr, and Pt. Asterisk indicates significant difference at *p* < 0.05, *t*-test, *n* = 6. Bars = 100 μm.

Feeding Sr^2+^ from fruit stalks, Sr was chiefly distributed in the phloem tissue in the pedicel especially around the cambium (**Figure 8**), which is similar to Ca distribution in the fruit stalk. Sr accumulation was also observed in the pericarp (**Figure 9A**). Girdling made on fruit stalk significantly reduced Sr abundance in the pericarp (**Figures 9B,C**). The result suggested this calcium analog was chiefly transported to fruit via the phloem pathway.

## Discussions

A constant supply of root-absorbed Ca to fruit is crucial for healthy fruit development. Long-distance transport of Ca is via xylem/apoplast pathways from root to top parts, which is chiefly driven by transpiration (Saure, 2005; Yang and Jie, 2005; Tonetto de Freitas and Mitcham, 2012; Hocking et al., 2016), and in case of fruit Ca uptake, fruit expansion is also a determinant for sap inflow that delivers Ca into fruit (Montanaro et al., 2015). However, studies have indicated that the route of Ca movement to fruit might not be a “smooth” one, as it is influenced by xylem hydraulic conductivity and pectin property of vessel cell wall (Ford and Quinlan, 1979; Hocking et al., 2016). In litchi, a by far higher Ca concentration was found in the pedicel than in the fruit pericarp (Huang et al., 2006; **Figure 2**) while in the fruit higher Ca concentrations were found in the more distal portions (**Figure 2**), reminiscent of “bottleneck effect” in Ca transport to fruit at the fruit end of the pedicel. In order to understand its causes, we need to understand the pathway of Ca transport.

The generally accepted idea is that Ca enters fruit chiefly through the xylem/apoplast pathway (Ferguson, 1980; Hanson, 1982; Zocchi and Mignani, 1995; Saure, 2005; Tonetto de Freitas and Mitcham, 2012; Hocking et al., 2016), and therefore fruit Ca uptake is believed to be determined by Ca content in the xylem sap and fruit transpiration and growth (Tonetto de Freitas and Mitcham, 2012; Hocking et al., 2016). Studies have shown Ca accumulation is closely related to xylem sap uptake by fruit, and the loss of xylem functionality especially in the late fruit development causes reduction in Ca uptake (Dichio et al., 2003; Miqueloto et al., 2014). An important reason to exclude the phloem/symplast pathway for Ca movement is the immobility of Ca in the pathway due to the limitation of a very low Ca concentration maintained in the symplastic system (Raven, 1977; Ferguson, 1980; Zocchi and Mignani, 1995; Saure, 2005; Tonetto de Freitas and Mitcham, 2012), which is believed to be maintained below 1 μM (Zocchi and Mignani, 1995; Hepler, 2005).

Despite the generally accepted idea of exclusive apoplastic Ca transport, pathway nature of nutrition Ca transport is still a matter of dispute. Studies have shown symplastic system may play a major role in delivery of Ca as a nutrition element. Cholewa and Peterson (2004) found radial movement of Ca from root surface to stele, upon which all Ca supply to the above-ground part depends, was almost entirely through the symplastic pathway that bypasses the apoplastic block of the Casparian bands in onion root. In apple seedling, the acropetal movement of ^45^Ca was shown to primarily through phloem pathway (Stebbins and Dewey, 1972). There are also studies showing that both the xylem and phloem participated in Ca movement into fruit (see reviews Himelrick and McDuffie, 1983; Yang and Jie, 2005). Jones et al. (1983) suggested that phloem might also be a major pathway for calcium transport to fruit after they found great difference between estimated calcium accumulation based on vessel influx volume and calcium concentration and measured calcium accumulation in apple fruit. Himelrick and McDuffie (1983) suggested that Ca transport into apple is likely via the phloem but movement of Ca inside fruit is through the xylem. Using various measuring methods, Brauer et al. (1998) found the concentration of Ca in phloem sap in a range of 10–100 μM, which is far higher than that reported in cytosol and even higher than commonly believed level below 1 μM (Zocchi and Mignani, 1995; Hepler, 2005). It seems that the phloem/symplast may be a more efficient route for Ca delivery than is commonly expected. There are some evidences indicating that Ca transport to fruit might not be an entirely passive apoplatic process. Volz et al. (1996) found that Ca accumulation in apple fruit was leaf-depended. Although this dependence was explained by Ca move-up driven by leaf transpiration, transpiration of the leaves directs xylem sap Ca to the leaves instead of fruit, and there is a lack of Ca mobility from leaves to fruit (Ferguson, 1980). Foliar application of ABA suppressed transpiration and promoted fruit Ca uptake in tomato (Tonetto de Freitas et al., 2011). Therefore, the positive role of leaves in Ca accumulation in fruit might not simply be related to transpiration. Bar-Tal et al. (1999) observed a positive correlation between Ca accumulation in apple fruit and carbohydrate concentration in the phloem, which indicates that photosynthates might play a role in Ca uptake in fruit. The association of acropetal movement of Ca and metabolism-driven basipetal movement of IAA (Banuelos et al., 1987; Cutting and Bower, 1989; Brown and Ho, 1993; Bangerth, 2000) indicates involvement of phloem/symplast in Ca transport. Ca in apex bud or fruit was reduced by girdling (Stebbins and Dewey, 1972; Arakawa et al., 1998; Vemmos, 2005; **Table 1**), which cuts the phloem pathway but not the xylem pathway. Although girdling induced reduction in calcium accumulation in fruit can be explained by root starvation that reduces nutrition uptake, root starvation hypothesis cannot explain some effects of girdling. For example, girdling at a distal position in a source-competing growing or bearing shoot, which causes negligible root starvation, significantly reduced Ca uptake in terminal organs (Vemmos, 2005; Wang et al., 2010; **Table 1**). The concurrent reductions in Ca uptake and fruit size caused by shoot girdling (**Table 1**) indicate a close correlation between fruit growth potential and Ca uptake capacity. The results agree with suggestion that Ca uptake in plant organs is demand-regulated and is associated with strength and metabolic activity (Ford and Quinlan, 1979; Cutting and Bower, 1989).

In addition, we obtained some more evidences that support phloem/symplast pathway as an important contributor to Ca accumulation in litchi fruit. First, Ca concentration in stems was always higher in the bark esp. phloem than in the xylem (Himelrick and McDuffie, 1983, Figure 4). Ferguson (1980) regarded bark/phloem as a Ca sink. Ca concentration displayed a pattern of “higher Ca in older stems” in the bark, whereas it was relatively constant in the xylem (**Figure 4**), indicating that Ca flux into the symplastic phloem/cortex from the apoplastic xylem sap occurs constantly. The presence of crystals in the phloem shows a considerable amount of Ca was sequestered as Ca oxalate in the phloem (**Figure 5**), enabling continuous accumulation of Ca in the tissue. Ca flux into a sieve element through various Ca channels has been well defined (Van Bel et al., 2011). La^3+^ is a strong Ca channel blocker (Lewis and Spalding, 1998; Demidchik et al., 2002). A second line of evidence from our study supporting the involvement of the phloem/symplast pathway in Ca transport to fruit came from the experiment with La^3+^ treatment. Blocking Ca channels with La^3+^ resulted in a significantly reduction (55.6%) in Ca accumulation in litchi fruit, especially in the pericarp (**Table 2**). The treatment also inhibited growth and pigmentation of fruit (**Table 2** and **Figure 6**), which again shows the close association between fruit growth and Ca uptake. Even with a smaller “diluting effect” due to reduced fruit growth, the Ca concentration in the pericarp was still lower in the La^3+^ treated fruit than in the control fruit. The results proved that Ca transport into fruit involved influx of Ca into symplast via Ca channels. Coinciding with the reduction in Ca accumulation in fruit, Ca concentrations at the fruit base and in the xylem of the pedicel increased while that in the phloem decreased by La^3+^ treatment (**Table 2** and **Figure 7**). The results suggest that the uptake of Ca by fruit depends on Ca influx from the apoplastic xylem to the symplastic phloem occurring at the fruit base and in the pedicel.

Our third line of evidence came from the experimental tracing of Sr^2+^, which is an analog to Ca^2+^ and has been widely used as a calcium tracer in plants (Laszlo, 1994; Storey and Leigh, 2004; Rosen et al., 2006). Girdling made on the fruit stalk significantly reduced the accumulation of Sr in the pericarp. The results showed that Sr^2+^ was transported to fruit pericarp chiefly through the phloem/symplast pathway (**Figures 8, 9**).

However, a major concern about the phloem/symplast pathway of Ca transport is the balance between Ca supply and its signaling function. White and Broadley (2003) stressed that the phloem/symplast Ca transport system should fulfill the demand of the shoot for Ca without compromising intracellular Ca signals. Indeed, phloem sap contains important signal transduction elements that respond to Ca elevation, such as Ca^2+^-dependent protein kinase (Avdiushko et al., 1997). Studies have shown the concentration of Ca in phloem sap falls in a range of 10–100 μM, which is higher than the elevated Ca concentration triggering signal transduction (Himelrick and McDuffie, 1983; Brauer et al., 1998). Brauer et al. (1998) suggested that Ca-regulated proteins in sieve-tube sap might respond only to relatively high Ca concentrations. Still, influx of Ca into phloem sap from xylem sap may trigger occlusion of sieve plates and stop phloem mass flow when the rise of Ca concentration exceeds a threshold value (Van Bel et al., 2011). A mechanism in the phloem to balance Ca influx is needed to prevent free Ca^2+^ from becoming high enough to trigger such a response. Vacuoles in the phloem cells provide a huge reservoir for sequestering Ca by forming Ca oxalate crystals, which were observed in large quantities in the phloem tissue of the pedicel (**Figure 5**). Formation of Ca oxalate in the pedicel might be an important means to balance Ca influx from the xylem to maintain Ca^2+^ concentration in the phloem sap below the threshold level that triggers sieve plate occlusion as suggested by Van Bel et al. (2011). In this sense, sequestering of Ca by oxalate in the phloem of the pedicels might be hypothesized to play a positive role in symplastic Ca transport to fruit.

At this stage, a hypothetic picture of the “bottleneck” of Ca transport to litchi fruit can be drawn. Ca flux into the symplast/phloem from the xylem/apoplast occurs constantly via Ca channels during its course to fruit, this translocation being more intensive in the pedicel than in the more distant fruit stalk. The Ca flowed into the phloem may move toward the fruit with the phloem mass flow. However, it may also trigger active pumping of Ca^2+^ into the vacuole, where Ca^2+^ is sequestered by forming oxalate in the phloem cells, as a mechanism to prevent signaling response. Therefore, phloem in the pedicel accumulates Ca while transporting it to the fruit. In the end, the pedicel had a high calcium content in the phloem tissue.

Litchi is an arillate fruit with unique structure. Its entire pericarp develops into the fruit skin, while the flesh or the aril initiates and grows later from the funicle (Huang, 2001). The findings from this study lure us to look into other types of fruits with different structures. Our preliminary results obtained from citrus, persimmon and loquat show that the pedicel has always a significantly higher Ca concentration than the fruit, and within pedicel Ca abundance is greater in the phloem than in the xylem (Song et al., 2014), suggesting the mechanism of Ca uptake found in litchi fruit might be universally present. Further studies are necessary to clarify Ca uptake in different types of fruits.

In summary, during the transportation in the apoplastic xylem, calcium constantly fluxes into symplastic phloem in stems of litchi. Ca is more distributed in the phloem, which might be an important pathway for calcium transport to fruit. The formation of calcium oxalate might be a balancing mechanism that maximizes calcium transport while preventing calcium signaling.

## Author Contributions

W-PS, WC, and J-WY performed the field experiments and lab analyses and contributed draft writing. H-CW and X-MH contributed the experimental design, research fund and critical revising of the manuscript.

## Conflict of Interest Statement

The authors declare that the research was conducted in the absence of any commercial or financial relationships that could be construed as a potential conflict of interest.
